# Does the Effect of Stress on Smartphone Addiction Vary Depending on the Gender and Type of Addiction?

**DOI:** 10.3390/bs13100810

**Published:** 2023-09-30

**Authors:** Wei Tu, Yangang Nie, Qingqi Liu

**Affiliations:** 1 School of Education, Guangzhou University, Guangzhou 510006, China; tuwei@e.gzhu.edu.cn; 2 Mental Health Education Center for College Students, Department of Student Affairs, Hunan University of Science and Engineering, Yongzhou 425199, China; 3 College of Education for the Future, Beijing Normal University, Zhuhai 519087, China

**Keywords:** stress, smartphone addiction, type of addiction, gender differences

## Abstract

Stress is closely associated with smartphone addiction. Nevertheless, there is a dearth of studies investigating the potential variation in the effect of stress on smartphone addiction based on the specific addiction type and gender. We conducted a cross-sectional questionnaire survey among 596 high school students. The results revealed that the effect size of stress on smartphone addiction varied across different types of addiction. The strongest relationship was observed between stress and social media addiction, followed by the relationship between stress and information acquisition addiction. Furthermore, gender played a significant moderating role in stress and three types of smartphone addiction. Specifically, stress was strongly associated with information acquisition addiction overall, with no significant gender differences observed. In contrast, stress exhibited a strong correlation with social media addiction, which was significantly more prevalent among females. On the other hand, game addiction and short-form video addiction were both strongly associated with stress, but showed significantly higher prevalence among males. This study enhances current research by offering supplementary insights into the correlation between stress and smartphone addiction, as well as exploring the potential implications of intervening in smartphone addiction.

## 1. Introduction

Smartphone addiction refers to a condition characterized by an excessive desire for and usage of smartphones, resulting in psychological and social dysfunction [1,2,3]. Research has unequivocally demonstrated the detrimental effects of smartphone addiction on both physical and mental health. Specifically, research has shown that smartphone addiction disrupts sleep quality [4,5] and increases the risk of conditions such as headaches and finger pain [6], thus impacting physical health. Additionally, smartphone addiction has been associated with reduced subjective well-being [7,8] and an elevated risk of anxiety and depression [9,10], exacerbating mental health concerns. Furthermore, research suggests that the detrimental impact of smartphone addiction is prevalent across different age groups, including children, adolescents, young adults, and middle-aged and elderly individuals [3,6,10,11].

Stress was a prominent predictor in both the development and exacerbation of smartphone addiction. Research has demonstrated that stress not only directly predicts adolescent smartphone addiction but also indirectly predicts it by weakening self-control [12]. Moreover, stress is significantly associated with smartphone addiction in both teenagers and young adults [12,13,14]. Additionally, stress plays a vital mediating role between certain risk factors, such as alexithymia and trait procrastination, and smartphone addiction [15,16]. However, despite existing studies providing preliminary evidence on the impact of stress on smartphone addiction, there are still two issues that require further clarification.

One area that necessitates further elucidation is the limited amount of research that explores whether the impact of stress on four types of smartphone addiction varies. Researchers have identified that smartphone addiction can be classified into four distinct types based on different behavioral characteristics: social media addiction, game addiction, information acquisition addiction, and short-form video addiction [17]. These four addictive behaviors all exhibit typical symptoms of smartphone addiction, but they are directed toward different Internet applications. Social media addiction refers to an excessive craving for and immersion in online social activities, whereas game addiction is characterized by an excessive craving for and obsession with online gaming. Information acquisition addiction involves an excessive craving for and engagement in activities related to information retrieval and browsing, while short-form video addiction refers to an excessive craving for, fascination with, and usage of short video applications. The Mobile Phone Addiction Type Scale (MPATS), developed based on these four addiction types, has demonstrated good reliability and validity [17]. Although previous studies have established a significant positive correlation between stress and general smartphone addiction [15,16], as well as specific types of smartphone addiction such as social media addiction [18] and short-form video addiction [19], there is still a lack of comparative research simultaneously examining multiple types of smartphone addiction. Investigating the influence of stress on different categories of smartphone addiction can help advance the development of more specific and targeted interventions for individuals who are particularly vulnerable to stress and have smartphone addiction.

The other aspect worthy of further investigation is whether the influence of stress on smartphone addiction varies among different gender groups. Previous research has indicated that compared to males, females tend to experience higher levels of stress [20,21,22]. Furthermore, females are also more prone to smartphone addiction in the face of certain negative factors [23,24,25]. These findings suggest that the association between stress and smartphone addiction may be more pronounced in females than in males. However, if we consider different types of smartphone addiction, it may result in relatively complex outcomes. The relationship between stress and one type of smartphone addiction might be stronger in females, while it could be stronger in males for another type of smartphone addiction. These specific research findings not only expand upon the general conclusions of previous studies on smartphone addiction but also facilitate gender-specific research and interventions for individuals who experience stress, thereby helping address smartphone addiction more effectively.

In summary, this study aimed to conduct a comprehensive analysis of the effect of stress on smartphone addiction. It also examined whether the predictive effect of stress on smartphone addiction varies depending on the type of addiction and gender. We hypothesized that stress would have significant effects on four types of smartphone addiction, with potential variation in the effect sizes. Furthermore, we anticipated that gender may moderate the impact of stress.

## 2. Methods

### 2.1. Participants

This study received approval from the ethics committee at the institution of the first author. All participants provided informed consent prior to their involvement. A total of 635 adolescents in Central China participated in our questionnaire survey. They provided responses pertaining to their stress levels, smartphone addiction, demographic information (gender and age), and duration of smartphone usage. The survey was completed by 596 adolescents in the classroom during regular school lessons. Their ages ranged from 12 to 19 years (*M* = 14.80, *SD* = 1.85). Of the 596 adolescents, 306 (51.3%) were male and 290 (48.7%) were female. Additionally, 364 (61.1%) adolescents were junior high school students, while 232 (38.9%) were senior high school students.

### 2.2. Measurements

#### 2.2.1. Stress

Stress was assessed using the Chinese version [26] of the Depression Anxiety Stress Scale (DASS-21) [27]. The DASS-21 is a condensed version of the DASS-42, and, therefore, the final score for each subscale was multiplied by two. Participants were instructed to rate the degree to which they experienced seven statements using a four-point scale (0 = does not apply to me, 3 = applies to me very much or most of the time). Cronbach’s α was 0.95.

#### 2.2.2. Smartphone Addiction

The study utilized the Mobile Phone Addiction Type Scale [17] to evaluate four categories of smartphone addiction. The scale consists of a total of 26 items, with six items dedicated to measuring social media addiction, six items for game addiction, seven items for information-seeking addiction, and seven items for short-form video addiction. Participants are required to rate each item on a five-point scale, where 1 indicates “almost never” and 5 indicates “almost always”. The reliability of the subscales was assessed using Cronbach’s α, with values of 0.93, 0.94, 0.91, and 0.95 for the social media addiction, game addiction, information acquisition addiction, and short-form video addiction subscales, respectively.

#### 2.2.3. Other Variables

The participants selected their gender (male or female) and subsequently provided their age and average daily duration of smartphone usage.

### 2.3. Analytic Strategies

An initial analysis was conducted to test the correlation between stress and smartphone addiction, as well as any gender differences in these variables. Afterward, a moderation analysis was conducted using the PROCESS macro for SPSS [28] to investigate whether gender plays a moderating role. Covariates, such as age and smartphone use time, were included to control for potential confounding effects on the results.

## 3. Results

### 3.1. Preliminary Analysis

The associations between the variables are presented in Table 1. Stress exhibited positive correlations with social media addiction (*r* = 0.48, *p* < 0.001), game addiction (*r* = 0.27, *p* < 0.001), information acquisition addiction (*r* = 0.43, *p* < 0.001), and short-form video addiction (*r* = 0.24, *p* < 0.001).

We conducted a Skewness and Kurtosis test to analyze the distribution of the data. The results indicated that all variables exhibited Skewness and Kurtosis values within the range of [−1.96, +1.96]. These findings suggest that the data can be characterized as approximately normally distributed. Therefore, the data is considered appropriate for conducting an independent samples *t*-test (see Table 2). Males demonstrated lower scores in stress (*t* = −4.00, *p* < 0.001) and social media addiction (*t* = −5.96, *p* < 0.001) compared to females. Conversely, males displayed higher scores in game addiction (*t* = 3.85, *p* < 0.001) and short-form video addiction (*t* = 3.67, *p* < 0.001) compared to females. However, no significant gender differences were observed in information acquisition addiction (*t* = −1.52, *p* = 0.13).

### 3.2. Testing for Gender Differences

**Figure 1:**
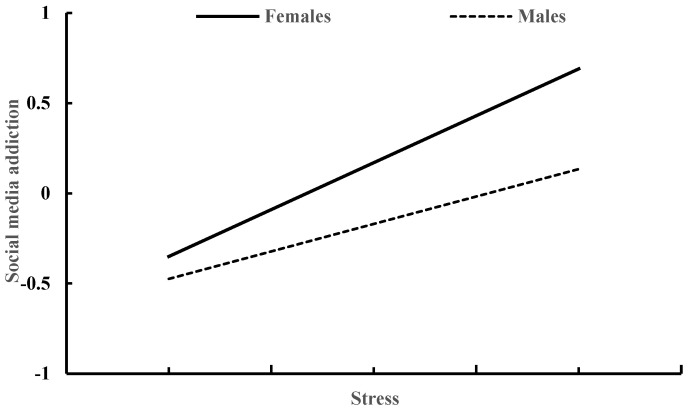
Stress and social media addiction in males and females.

Table 3 shows how gender moderates the relationship between stress and social media addiction. Controlling for age and smartphone use time, both stress (β = 0.42, *p* < 0.001) and gender (β = 0.34, *p* < 0.001) exhibited a positive association with social media addiction. Furthermore, the interaction between stress and gender was also found to have a positive impact on social media addiction (β = 0.22, *p* < 0.001). The correlation between stress and social media addiction was stronger in females (β = 0.53, *p* < 0.001) compared to males (β = 0.31, *p* < 0.001), as depicted in Figure 1.

**Figure 2:**
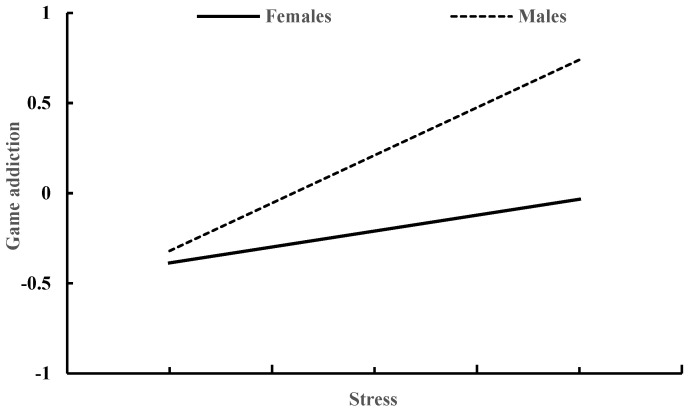
Stress and game addiction in males and females.

Table 4 shows how gender moderates the relationship between stress and game addiction. Controlling for age and smartphone use time, both stress (β = 0.36, *p* < 0.001) and gender (β = −0.42, *p* < 0.001) exhibited a significant association with game addiction. Furthermore, the interaction between stress and gender was also found to have a significant impact on game addiction (β = −0.36, *p* < 0.001). The correlation between stress and game addiction was stronger in males (β = 0.53, *p* < 0.001) compared to females (β = 0.17, *p* < 0.001), as depicted in Figure 2.

**Figure 3:**
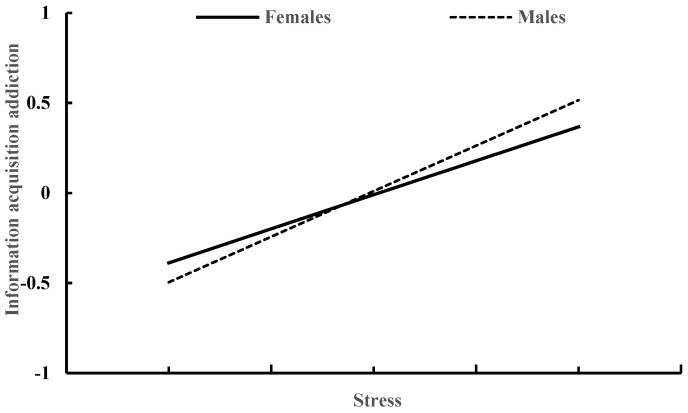
Stress and information acquisition addiction in males and females.

Table 5 shows how gender moderates the relationship between stress and information acquisition addiction. Controlling for age and smartphone use time, stress (β = 0.45, *p* < 0.001) displayed a positive association with information acquisition addiction. However, the effect of gender on information acquisition addiction was not significant (β = −0.02, *p* = 0.82). Additionally, the interaction between stress and gender did not have a significant impact on information acquisition addiction (β = −0.13, *p* = 0.17). These results indicate that gender did not significantly moderate the association between stress and information acquisition addiction, as illustrated in Figure 3.

**Figure 4:**
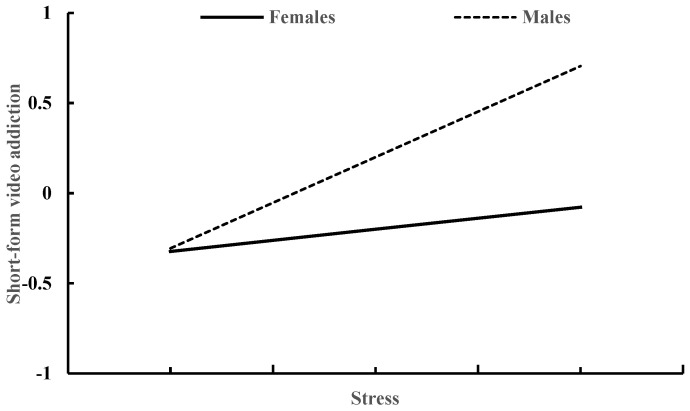
Stress and short-form video addiction in males and females.

Table 6 shows how gender moderates the relationship between stress and social media addiction. Controlling for age and smartphone use time, both stress (β = 0.32, *p* < 0.001) and gender (β = −0.40, *p* < 0.001) exhibited a significant association with short-form video addiction. Furthermore, the interaction between stress and gender was also found to have a significant impact on short-video addiction (β = −0.39, *p* < 0.001). The correlation between stress and short-form video addiction was stronger in males (β = 0.51, *p* < 0.001) compared to males (β = 0.12, *p* < 0.001), as depicted in Figure 4.

## 4. Discussion

While numerous studies have been conducted on smartphone addiction, very few have comprehensively analyzed and compared various types of smartphone addictions. This particular study focused on examining the correlation between stress and smartphone addiction, aiming to build upon previous research. Specifically, the study investigated whether the association between stress and smartphone addiction differs based on the type of addiction. Additionally, the study explores whether gender moderated the effect of stress on smartphone addiction. The results showed that the intensity of the relationship between stress and smartphone addiction varies based on the type of addiction. Furthermore, gender played a role in moderating the effect of stress on three types of smartphone addiction. The results can carry significant implications for the development of tailored interventions that address the experience of stress among male and female students in different groups, with the ultimate objective of mitigating smartphone addiction and minimizing problematic behaviors.

This study revealed that stress had a significant impact on all four types of smartphone addiction, corroborating previous research on the association between stress and general smartphone addiction [12,13,14,15,16]. However, the effect size of stress on smartphone addiction varied across different types of addiction. More specifically, the strongest relationship was found between stress and social media addiction, followed by the relationship between stress and information acquisition addiction. In contrast, the effects of stress on game addiction and short-form video addiction were less pronounced. The findings demonstrate that when faced with stress, individuals are prone to excessively engage in mobile socializing or information browsing. Some may seek solace in social activities as a means of stress relief [29], while others may turn to information retrieval to better manage stressful situations [30]. Social activities offer individuals emotional support, and smartphones conveniently cater to various social needs. Consequently, individuals may excessively depend on their smartphones for social activities to attain emotional support when confronted with stressful situations [31,32]. However, the abundance of interpersonal relationships on social media platforms can lead to individuals becoming overly engrossed in mobile socializing and eventually developing an addiction. Similarly, information-seeking behaviors can provide individuals with informational support, and mobile phones serve as a convenient tool for satisfying their need to retrieve and obtain information. Consequently, many individuals may become overly dependent on searching and browsing relevant information as a means to address stress-inducing events [33,34]. However, the vast array of information available through various mobile applications can cause individuals to become lost in an overwhelming sea of knowledge and develop an addiction to information acquisition. Additionally, short videos [17,35] and mobile games [36,37] have emerged as coping mechanisms for some individuals in response to stress. However, the findings of the present study indicate that in the case of high-stress populations, it is imperative to prioritize the examination of social media addiction and information addiction.

In addition, this study revealed significant gender differences in three types of mobile phone addiction, with the exception of information acquisition addiction. Specifically, there was no clear disparity between males and females in terms of information acquisition addiction. However, females demonstrated notably higher levels of social media addiction compared to males, whereas males exhibited significantly higher levels of gaming and short-form video addiction compared to females. These findings provide an explanation for the inconsistent research findings regarding gender differences in smartphone addiction and contribute to a better understanding of the complex gender differences that exist in smartphone addiction when considering multiple types of addictions. The analysis of gender differences in smartphone addiction should be based on specific addictive behaviors. Different types of addictive behaviors may show distinctly different gender differences.

Based on the gender differences in specific types of smartphone addiction, this study also revealed a gender-based moderation effect on the relationship between stress and three types of smartphone addiction. Specifically, the association between stress and social media addiction was stronger in females compared to males. On the contrary, there was a stronger correlation between stress and both game addiction and short-form video addiction in males compared to females. These findings suggest that the interplay between addiction types and gender plays a crucial role in shaping the association between stress and smartphone addiction. Previous research has consistently identified significant gender differences in social media addiction, with females displaying higher levels of addiction compared to males [38,39]. Females are more susceptible to excessive phone use for social interaction when confronted with negative life events [23,39]. This propensity may stem from their heightened need for relatedness and a strong inclination to seek social support and emotional release through interpersonal communication [40,41]. However, it is essential to acknowledge that online social interactions only offer temporary relief for negative emotions, and repeated exposure to diverse stressors may drive females to excessively rely on social media activities, ultimately leading to social media addiction. Conversely, males tend to employ emotional inhibition [20] and avoidance coping in response to stress [42]. They are less inclined than females to seek support from social relationships [41,42] and may instead cope with stress through mobile gaming or engaging with short videos. As a result, males are more susceptible to developing mobile gaming addiction and short video addiction compared to females. It is worth noting that there is no significant difference between genders in the relationship between stress and information acquisition addiction. Both males and females are susceptible to developing information addiction in the presence of stress. This finding suggests that individuals of both genders may excessively rely on their phones to search and browse information, using them as a means to analyze and solve problems when faced with stress. However, according to the strength model of self-control and relevant empirical studies [43,44,45], stress depletes an individual’s limited psychological resources, resulting in a decline in self-control, which hampers the processes of autonomous decision making and behavior. In such circumstances, individuals may become increasingly reliant on Internet information, eventually developing information addiction.

This study provides two valuable insights for the prevention of and intervention in smartphone addiction. Firstly, it is crucial to pay special attention to social media addiction and information acquisition addiction, particularly among individuals who are sensitive to stress, such as children and adolescents with immature self-control abilities. In times of stress, these two forms of addiction tend to be particularly strong in adolescents. Therefore, it is important for schools and parents to guide adolescents with high academic stress, discouraging excessive use of online socializing and information seeking as a means to alleviate stress. Secondly, it is important to tailor addiction interventions based on gender-specific patterns. While stress-induced information addiction does not exhibit significant gender differences, girls tend to display more severe addiction to social media when faced with stress, while boys demonstrate more severe addiction to Internet games and short-form videos. Therefore, schools and parents can guide girls to seek support from parents and teachers through real-life interactions as a coping strategy for stress, and encourage boys to alleviate stress through physical exercise, participation in school clubs, or engagement in class activities.

There are two primary limitations in this study. Firstly, it utilized a cross-sectional survey design, which does not allow for the establishment of a strict causal relationship between stress and smartphone addiction. Future research should consider utilizing experimental designs to manipulate stress levels and examine potential variations in smartphone addiction levels. Secondly, given that our study was conducted among Chinese adolescents, it is important to approach the generalization of our findings to other adolescent populations in different countries with caution. Future research endeavors could investigate and compare adolescents from various countries and cultural backgrounds.

## 5. Conclusions

Previous studies have confirmed the association between stress and smartphone addiction. However, there is a lack of research specifically examining the influence of addiction type and gender on this relationship. Therefore, this study aimed to analyze and compare the relationship between stress and different types of smartphone addiction while also investigating whether gender acts as a moderating factor. The results indicated that stress significantly predicted all four types of smartphone addiction. However, the magnitude of this relationship varied across the different types of addiction. Furthermore, gender was found to significantly moderate the effect of stress on three types of smartphone addiction. This study underscores the importance of examining the relationship between stress and smartphone addiction by considering different types of addiction and gender groups. Moreover, it provides useful insights for targeted prevention and intervention strategies aimed at addressing smartphone addiction among adolescents.

## Figures and Tables

**Table 1:** Intercorrelations between variables.

Variables	*M*	*SD*	1	2	3	4	5
1. Stress	1.27	0.98	—				
2. Social media addiction	2.52	1.25	0.48 ***	—			
3. Game addiction	2.28	1.08	0.27 ***	0.34 ***	—		
4. Information acquisition addiction	2.19	1.02	0.43 ***	0.49 ***	0.50 ***	—	
5. Short-form video addiction	2.12	1.07	0.24 ***	0.43 ***	0.50 ***	0.42 ***	—

Note. *** *p* < 0.001.

**Table 2:** Gender differences in smartphone addiction.

Variables	Group	*M*	*SD*	*t*	*p*
Stress	Males	1.11	0.81	−4.00	<0.001
	Females	1.43	1.11		
Social media addiction	Males	2.26	1.11	−5.96	<0.001
	Females	2.82	1.31		
Game addiction	Males	2.46	1.17	3.85	<0.001
	Females	2.11	0.94		
Information acquisition addiction	Males	2.13	1.05	−1.52	0.13
	Females	2.25	0.99		
Short-form video addiction	Males	2.27	1.16	3.67	<0.001
	Females	1.96	0.94		

**Table 3:** Moderation of gender in the association between stress and social media addiction.

Regression Equation		Significance of Regression Coefficients				Bootstrap	
Outcome	Independent Variables	β	SE	*t*	*p*	LLCI	ULCI
Social media addiction	Constant	−0.02	0.04	−0.50	0.62	−0.09	0.05
	Age	0.03	0.04	0.80	0.42	−0.04	0.10
	smartphone use time	0.04	0.03	1.26	0.21	−0.02	0.11
	Gender	0.34 ***	0.07	4.69	<0.001	0.20	0.49
	Stress	0.42 ***	0.04	10.02	<0.001	0.33	0.50
	Stress × Gender	0.22 **	0.08	2.72	<0.01	0.06	0.39

Note. Bootstrap sample size = 5000. SE = standard error. LL = low limit, CI = confidence interval, UL = upper limit. ** *p* < 0.01, *** *p* < 0.001. The same applies to the tables below.

**Table 4:** Moderation of gender in the association between stress and game addiction.

Regression Equation		Significance of Regression Coefficients				Bootstrap	
Outcome	Independent Variables	β	SE	*t*	*p*	LLCI	ULCI
Game addiction	Constant	0.03	0.04	0.74	0.46	−0.05	0.11
	Age	0.02	0.04	0.44	0.66	−0.06	0.10
	Smartphone use time	0.01	0.03	0.04	0.97	−0.06	0.06
	Gender	−0.42 ***	0.08	−5.32	<0.001	−0.58	−0.27
	Stress	0.36 ***	0.05	7.38	<0.001	0.26	0.45
	Stress × Gender	−0.36 ***	0.10	−3.77	<0.001	−0.55	−0.17

Note. Bootstrap sample size = 5000. SE = standard error. LL = low limit, CI = confidence interval, UL = upper limit. *** *p* < 0.001.

**Table 5:** Moderation of gender in the association between stress and information acquisition addiction.

Regression Equation		Significance of Regression Coefficients				Bootstrap	
Outcome	Independent Variables	β	SE	*t*	*p*	LLCI	ULCI
Information acquisition addiction	Constant	0.01	0.04	0.27	0.79	−0.07	0.09
	Age	0.04	0.04	0.94	0.35	−0.04	0.11
	Smartphone use time	−0.04	0.03	−1.28	0.20	−0.10	0.02
	Gender	−0.02	0.08	−0.23	0.82	−0.17	0.14
	Stress	0.45 ***	0.05	9.43	<0.001	0.36	0.55
	Stress × Gender	−0.13	0.09	−1.37	0.17	−0.32	0.06

Note. Bootstrap sample size = 5000. SE = standard error. LL = low limit, CI = confidence interval, UL = upper limit. *** *p* < 0.001.

**Table 6:** Moderation of gender in the association between stress and short-form video addiction.

Regression Equation		Significance of Regression Coefficients				Bootstrap	
Outcome	Independent Variables	β	SE	*t*	*p*	LLCI	ULCI
Short-form video addiction	Constant	0.03	0.04	0.78	0.44	−0.05	0.11
	Age	0.02	0.04	0.44	0.66	−0.06	0.09
	Smartphone use time	−0.03	0.03	−0.93	0.36	−0.10	0.03
	Gender	−0.40 ***	0.08	−4.85	<0.001	−0.56	−0.24
	Stress	0.32 ***	0.05	6.38	<0.001	0.22	0.42
	Stress × Gender	−0.39 ***	0.10	−3.91	<0.001	−0.59	−0.20

Note. Bootstrap sample size = 5000. SE = standard error. LL = low limit, CI = confidence interval, UL = upper limit. *** *p* < 0.001.

## Data Availability

Data are available on reasonable request from the corresponding author.
